# Treatment of Addison's Disease

**Published:** 1903-10-10

**Authors:** 


### TREATMENT OF ADDISON'S DISEASE.

Although the results of organotherapy in Addison's disease are not nearly so good as those obtained by similar treatment in affections of the thyroid gland, yet it appears that better results can be obtained by the administration of fresh supra-renal gland or of the extract than by any other method which has been at all extensively tried. This seems to be clearly proved by a very useful summary of 97 cases treated by organotherapy, of which Dr. E. W. Adams[1] has collected details. As is well known, the course of Addison's disease is usually characterised by marked variations; at one time there will be distinct improvement of the patient's condition, only to be followed by an advance of the disease to a stage it has not reached before. This being the case it is not possible to draw reliable deductions as to the effect of any particular treatment from single cases. Hence the value of a large collection such as Dr. Adams has made. Analysing the 97 cases he finds that seven were made distinctly worse, 43 derived no real benefit, 31 showed marked improvement, and 16 were permanently relieved. With regard to the way in which the administration was performed, Dr. Adams gives a table in which he classifies the results obtained by the following methods:—(1) Subcutaneous grafts of the supra-renal glands of dogs (three cases, all of which ended fatally within three days); (2) administration of fresh gland by the mouth (nine cases, of which only two were unimproved); (3) hypodermic or intra-muscular injections (11 cases, seven of which showed no improvement. In some cases the injections caused great pain). (4) Fluid or solid extract given by mouth (61 cases, of which 34 were unimproved); (5) mixed methods. (Five cases are given where the drug was given hypodermically and by the mouth, and all of these cases were improved). (5) Unclassed. Dr. Adams thinks it may be safely concluded from these results that there are cases of Addison's disease which undoubtedly derive benefit from treatment with some form of supra-renal substance, though in any particular case it is not possible to predicate the likelihood of success or failure. Some of the successful cases mentioned in his tables were in a very advanced stage of the disease at the time when the treatment was begun. In others where no improvement was obtained administration of supra-renal substance had been commenced at quite an early stage. An important disturbing factor in all the cases is the difficulty in estimating the effect of other treatment, such as rest and general hygienic measures, carried out simultaneously with the "specific" treatment, but it is a significant fact in considering this point to note that before the advent of organo-therapy it was extremely rare for a case of Addison's disease to apparently recover. Moreover, among the cases brought forward and analysed by Dr. Adams there are several cases among those returned as improved, which showed definite relapses when the drug was discontinued for a time and which improved so soon as the treatment was resumed. The best method of administering the drug would appear to be to give the fresh gland substance, or failing this an extract of the gland, by the mouth.

[1] Practitioner, October, 1903.

